# Current state and future directions of technology-based ecological momentary assessments and interventions for major depressive disorder: protocol for a systematic review

**DOI:** 10.1186/s13643-018-0899-y

**Published:** 2018-12-13

**Authors:** Desirée Colombo, Azucena Garcia Palacios, Javier Fernandez Alvarez, Andrea Patané, Michelle Semonella, Pietro Cipresso, Marta Kwiatkowska, Giuseppe Riva, Cristina Botella

**Affiliations:** 1 Department of Basic Psychology, Clinic and Psychobiology, Universitat Jaume I, Av. Sos Baynat, s/n, 12071 Castellón, Spain; 2 Applied Technology for Neuro-Psychology Lab, IRCCS Istituto Auxologico Italiano, Via Magnasco, 2, 20149 Milan, Italy; 3 Department of Psychology, Università Cattolica del Sacro Cuore, Largo Gemelli, 1, 20100 Milan, Italy; 4 Department of Computer Science, University of Oxford, Wolfson Building, Parks Rd, Oxford, OX1 3QD UK; 5 CIBER Fisiopatología Obesidad y Nutrición (CIBERobn), Instituto Salud Carlos III, Madrid, Spain

**Keywords:** Ecological momentary assessment, Ecological momentary intervention, Major depressive disorder, mHealth

## Abstract

**Background:**

Ecological momentary assessments (EMAs) and ecological momentary interventions (EMIs) represent a novel approach for the assessment and delivery of psychological support to depressed patients in daily life. Beyond the classical paper-and-pencil daily diaries, the more recent progresses in Information and Communication Technologies (ICT) enabled researchers to bring all the needed processes together in only one device, i.e., response signaling, repeated symptom collection, information storage, secure data transfer, and psychological support delivery. Despite evidence showing the feasibility and acceptability of these techniques, EMAs are only beginning to be applied in real clinical practice, whether the development of EMIs for clinically depressed patients is still very limited. The objective of this systematic review is to provide the state of the art of technology-based EMAs and EMIs for major depressive disorder (MDD), with the aim of leading the way to possible future directions for the clinical practice.

**Methods:**

We will conduct a systematic review using the Preferred Reporting Items for Systematic Reviews and Meta-Analysis (PRISMA) guidelines. Data sources will include two bibliographic databases, PubMed and Web of Science (Web of Knowledge), supplemented by searches for unpublished or ongoing studies. Eligible studies will report data for adult (≥ 18 years old) with a primary (both current and past) diagnosis of MDD, defined by a valid criterion standard. We will consider studies adopting technology-based EMAs and EMIs for the investigation and/or assessment of depression and for the delivery of a psychological intervention. We will exclude studies adopting paper-and-pencil tools.

**Discussion:**

The proposed systematic review will provide new insights on the advantages and benefits of adopting technology-based EMAs and EMIs for MDD in the traditional clinical practice, taking into consideration both clinical and technological issues. The potential of using sensors and biosensors along with machine learning for affective modeling will also be discussed.

**Electronic supplementary material:**

The online version of this article (10.1186/s13643-018-0899-y) contains supplementary material, which is available to authorized users.

## Background

### Rationale

Major depressive disorder (MDD) is one of the leading causes of disease and disability in the world, affecting approximately the 4.4% of the general population [1]. Other than impairing daily functioning [2] and quality of life [3], convergent evidence indicates that depression is often associated with high rates of non-recovery, recurrence, and comorbidity [4, 5]. Only in the USA, the economic burden associated with depression was estimated at $83.1 billion in 2000, at $173.2 billion in 2005, and at $210.5 billion in 2010 [6]. These incremental costs represent an important public health issue, posing the clinical field with the challenge of developing new efficacious ways to assess and deliver psychological support to depressed people. In that sense, Information and Communication Technologies (ICTs) have the potential to improve traditional clinical tools, as we will discuss in the following paragraphs.

### Technology-based ecological momentary assessments for depression

In clinical practice, symptom assessment mainly relies on the use of retrospective questionnaires and self-reports [7]. On the one hand, a growing number of studies pointed out the existence of multiple factors affecting mood recall [8] and, especially in depressed patients, the presence of a general recall bias [9, 10], like increased elaboration of negative details, difficulties in disengaging from negative material, and greater recall of negative rather than positive events [11, 12]. On the other hand, symptom fluctuations over time or even across one single day [13, 14] may lower the accuracy and reliability of traditional assessments, thus suggesting the importance of capturing symptom dynamics with higher precision [15, 16].

In the last decade, ecological momentary assessments (EMAs) received increasing interest and attention. According to Csikszentmihalyi, EMAs represent “an attempt to provide a valid instrument to describe variations in self reports of mental processes” and allow therefore to investigate affect, thoughts, behaviors, and symptom fluctuations over time and, importantly, during the flow of daily experience [7, 15, 17, 18]. Beyond the first studies adopting paper-and-pencil daily diaries [19], the use of electronic tools enabled researchers to bring all the needed processes together in only one device (i.e., response signaling, symptom collection, information storage, immediate feedbacks, secure data transfer). Additionally, it is nowadays possible to “indirectly” collect passive data by means of device-embedded sensors or wearable biosensors and to combine this information with self-reports [20]. Accordingly, the hierarchical sensing model proposed by Mohr underlines the great revolution that sensors and biosensors can bring, allowing to collect raw sensor data that can be converted in “behavioral markers” [21]. Furthermore, another potentiality relies in the application of machine learning algorithms. Patient-specific models can be automatically learnt that continuously estimate patients’ affective state [22]. Alternatively, predictive models that combine information from physiological and behavioral signals to estimate the patient’s future mood, stress level, and self-reported health (as for one or few days in advance) can be automatically inferred from patients’ data.

### Ecological momentary interventions for depression

Another challenge within the clinical field is the dissemination problem, highlighted by the great amount of people affected by mental disorders that are not receiving adequate treatments [23]. Despite the effectiveness of traditional face-to-face treatments [24], individual psychotherapy is not likely to be the solution for this large need [25].

Similarly to EMAs, ecological momentary interventions (EMIs) are an innovative way to deliver psychological support on hand-held mobile technologies during everyday life [26]. EMIs can be used either as stand-alone interventions or in association with other treatments [26], and with or without the involvement of a real therapist [27]. Thanks to the integration of self-reports with contextual and physiological data derived from embedded sensors or wearable biosensors, EMIs permit to develop context-aware systems and more personalized interventions [28]. Implemented along with short-term (hours) or medium-term (days) data-driven predictive models of patients’ mood, stress, or affective state, the continuous monitoring of data through sensors and biosensors could allow both for just-in-time interventions, and for intervention planning (medium-term) before the patient reaches a critical situation, eventually involving the therapist in the treatment loop.

### Previous systematic reviews

To our knowledge, no systematic review addressing the use of ecological momentary procedures by mean of electronic devices has been carried out.

Aan Het Rot et al. conducted a systematic review on the use of EMA techniques for the investigation of mood dysregulation [15]. Nevertheless, authors included studies with both MDD and bipolar disorder (BD) patients, and their main focus was on the obtained outcomes, but not on the method itself. In the same way, Ebner-Premier and colleagues conducted a systematic review on EMAs in the field of mood disorders and mood dysregulation, thus including MDD, BD, and borderline personality disorder (BPD) patients [16] to show how EMAs can better address research questions compared to laboratory or questionnaire studies. In both reviews, however, the adoption of technologies was not an inclusion criterion and most of the included studies were based on paper-and-pencil daily diaries. Regarding interventions, no systematic review has been conducted on EMIs for clinically depressed patients. To date, we could retrieve only a brief review investigating the use of this type of interventions among all types of psychiatric patients [29].

In conclusion, this is the first systematic review that specifically focuses on MDD patients and that concurrently investigate, both from a clinical and technological point of view, the adoption of electronic tools, sensor and biosensors for the development of EMAs and EMIs.

### Objective

To date, EMAs have been already adopted to investigate various mental disorders [15, 30–32]. Similarly, some EMIs have been created for schizophrenia, bipolar disorder and depression [30]. Nevertheless, there is still a very huge gap between the clinical practice and the research field [33]: Despite evidence supporting its feasibility and utility [16, 34, 35], the use of EMAs in clinical settings is still very scant and the development of EMIs for depression still very limited. On the contrary, the clinical field could significantly benefit from them for different reasons: (1) To overcome the dissemination problem and develop new tools that could increase the number of reachable people, (2) to create novel assessments and interventions for MDD patients, giving much more importance to the ecological and momentary aspects, and increasing the precision of clinical assessments, (3) to develop more customized interventions, mainly thanks to the potential of machine learning techniques applied to data from sensors or biosensors.

The objective of this review is therefore to provide the state of the art of technology-based EMAs and EMIs for MDD, with the aim of leading the way to possible future directions for the clinical practice. To realize this, our review will explore the current literature on EMA and EMI for depression, with a detailed analysis of the technological aspects, clinical outcomes, advantages, and challenges.

The main exploratory research question of this systematic review will be the following:

- Which are the technological characteristics (devices, sensors, biosensors) and the clinical features and outcomes (fields of application, sampling schemas, compliance, dropout rates, results obtained) of the available EMAs and EMIs for depression?

In light of the obtained results, the discussion paragraph will aim at clarifying these two explorative points:

- How and why could the clinical practice benefit from the use of EMAs and EMIs?
- Building upon recent advances in machine learning for affective modeling and on the available examples of EMA and EMI studies adopting these techniques, what are the current gaps and future developments of EMAs and EMIs that could be tackled thanks to the combined use of sensors and biosensors data in addition to self-reports?

## Methods

We will follow the Preferred Reporting Items for Systematic Reviews and Meta-Analysis (PRISMA) guidelines [36]. The PRISMA-P checklist document shows this in more detail (see Additional file 1).

### Eligibility criteria

To formulate research questions and facilitate literature search, the PICO framework has been followed [36].

### Participant characteristics (P)

We will include studies involving a sample of adults (≥ 18 years old and < 65 years old) with a primary (both current and past) diagnosis of MDD diagnosed using any recognized diagnostic criteria.

### Study characteristics (I)

#### Ecological momentary assessment

We will include studies adopting an ecological momentary assessment by means of hand-held technologies (such as smartphones, personal digital assistants, or hand-held computers) for the collection of daily self-reports. The daily self-assessments can be composed of sets of free items or a standardized questionnaire (for instance, daily administration of the PHQ-9). We will not include EMAs that only rely on the collection of passive data from sensors and biosensors. Indeed, we think that self-reports play a key role within this approach, as objective observations do not always reflect the subjective experience of them [37]. Accordingly, a proper clinical assessment should always consider also patients’ subjective self-reports and experienced emotional states. However, we will include studies combining self-reports with data gathered from wearable biosensors or device-embedded sensors, and applying machine learning techniques to the acquired data, when available.

There are no restrictions in relation to context, such as geographical location, cultural factors, or language of the assessment/intervention.

#### Ecological momentary intervention

We will include “momentary” interventions that are provided on a hand-held technology during patients’ daily life and in specific moments of the day, i.e., according to patients’ real-time needs. The included interventions can be either a stand-alone or adjunctive intervention. As for EMAs, we will also include EMIs collecting information from wearable biosensors or device-embedded sensors and applying machine learning techniques to the acquired data. There are no restrictions in relation to context, such as geographical location, cultural factors, or language of the assessment/intervention.

### Comparators (C)

As we are interested in investigating all attempted developments and applications of EMI and EMA for MDD, there are no restrictions in terms of comparators.

### Outcome measures (O)

#### Ecological momentary assessment

As we are interested in the ecological method itself (i.e., technology-based EMAs), we will include studies applying EMAs both for clinical assessments and research purposes. Therefore, there are no restrictions with regard to outcome measures. We will focus both on technological aspects (type of device, adoption of sensors or biosensors, and addition to self-reports) and clinical outcomes (investigated variables, compliance, dropout).

#### Ecological momentary intervention

The primary outcome measure will be clinical improvement, defined as the reduction of depressive symptoms at the end of the proposed intervention. If available, the following secondary outcome measures will also be considered: compliance and dropout rates.

### Type of studies

We will include only English papers and articles that have an available full-text. Moreover, we will exclude the following types of manuscripts: conference papers, reviews, notes, case reports, letters to the editor, editor’s notes, extended abstracts, proceedings, patents, editorials, and other editorial materials. There are no limitations with respect to the design of assessment and intervention studies to be eligible for inclusion; therefore, also non-randomized controlled trials will be considered.

### Searching and selection process

#### Preliminary search

A preliminary literature search in PubMed was performed using key terms related to MDD and EMA/EMI. The retrieved articles were used to identify further keywords and build an adequate search string.

### Search strategy

According to the preliminary search, we will use the combination of terms listed in Table 1. Medical subject headings (MeSH) or equivalent and text word terms will be used. The search will be performed in the following electronic databases: PubMed and Web of Science (Web of Knowledge). Information sources will not be restricted to a specific time period. We will also search for unpublished or ongoing researches that could be of interest for this review throughout bibliographies of the retrieved studies, and by asking to experts of the field.

**Table 1:** Search terms to be used in the search strategy

Concept	Search terms
EMA–EMI	*EMA, ecological momentary assessment, EMI, ecological momentary intervention, mobile health, mHealth, mobile phone, smartphone, ecological momentary intervention, ESM, experience sampling method, ambulatory assessment, personal digital assistant, ambulatory monitoring, real time data capture, real time monitoring, real time interventions, computer assisted diary, electronic diary*
Major depressive disorder	Depression, MDD, major depressive disorder, major depression, unipolar depression, emotion dysregulation, affective disorder, mood disorder, depress*, affective symptoms, depressive symptoms

ESM experience sampling method

### Selection process

To identify and delete duplicates, database search will be imported to a reference management software (Endnote ×8). One author (D.C.) will further screen outcomes manually for other duplicates.

Studies will be independently selected by three authors (D.C., M.S., and J.F.A.). First, the three authors will screen titles and abstracts of the retrieved studies, excluding manuscripts that are not relevant for this systematic review. The reasons for rejection will be annotated. Subsequently, authors will retrieve the full text-copies of the remaining articles and select those meeting the inclusion criteria. Disagreements will be resolved through discussion and consensus, involving a fourth author (C.B.) if necessary. The proportion of agreement will be presented in the final review. The PRISMA template will be used to reproduce the flow chart with details on the selection process [38].

### Data extraction

Three authors (C.D., J.F.A., and M.S) will create an Excel data sheet and extract data from the selected studies. Studies will be divided in two categories: EMA studies and EMI studies.

#### Ecological momentary assessment

Data extracted from EMA studies will be as follows:

- General: authors, article title, type of publication, year of publication;
- Study characteristics: aim of the study, main variables of interest, type of electronic device, adoption of sensors and biosensors, sampling methods, assessment duration, and type of data analysis (i.e. use of machine learning techniques);
- Participants: number of participants, type of control group, inclusion/exclusion criteria, dropout rates;
- Outcome measures: unit of measurement, type of measurement, primary outcomes, compliance rates.

#### Ecological momentary intervention

Data extracted from EMI studies will be the following:

- General: authors, article title, type of publication, year of publication;
- Study characteristics: aim of the study, type of electronic device, adoption of sensors and biosensors, sampling methods, duration and intensity of the treatment, type of data analysis (i.e., use of machine learning techniques);
- Participant: number of participants, control group, inclusion/exclusion criteria, dropout rates;
- Outcome measures: unit of measurement, type of measurement, successfulness of the intervention, compliance, participants’ satisfaction.

Using the aforementioned key items, authors will identify and extract data independently. Subsequently, data will be compared, and any divergence will be solved by discussion, involving a fourth author if necessary (C.B.).

### Quality assessment of the included studies

#### Ecological momentary assessment

The first aim of this review will be to describe the available technology-based EMAs for the assessment of MDD and the investigation of its clinical manifestation, taking into consideration both technological issues and fields of application. Therefore, the assessment of methodological bias is not planned for the selected EMA studies. Authors will however analyze results taking into consideration the methodological quality and study designs of the selected studies.

#### Ecological momentary intervention

The primary outcome measure will be the efficacy of the proposed intervention. As both randomized and non-randomized controlled trials will be included, the risk of bias will be assessed with the Downs and Black Quality Index [39], providing both an overall score for study quality and single scores for quality of reporting, internal validity, power, and external validity. Two reviewers (D.C and J.F.A.) will independently assess the methodological quality of the included studies.

### Data synthesis and result presentation

Two authors (D.C. and J.F.A.) will systematically read the selected studies and analyze the results of each of them. The obtained synthesis will be regularly discussed and shared with the other authors. Results will be summarized in two different tables (Table 2) and better described in a narrative way within the result paragraph that will be divided in two sub-sections: EMA studies and EMI studies.

**Table 2:** Key items for result tables

	Key items
EMA	*Author(s), Sample(s), Variable(s), Device(s), Sensor(s), Duration, Prompt(s) per day, Sampling Schema, Primary Outcome(s);*
EMI	*Author(s), Name of the intervention, Sample(s), Content of the Intervention, Duration, Sensor(s), Primary Outcome(s).*

#### Ecological momentary assessment

We will first provide an accurate description of the psychological fields of application of EMA techniques, identifying for each of them the advantages derived from this approach. Subsequently, we will deepen the technological specifications (i.e., type of electronic devices, sensors, biosensors), design features (i.e., sampling schemas, duration, number of prompts), and clinical outcomes (i.e., obtained results, compliance, and dropout rates) for each included study.

#### Ecological momentary interventions

We will first provide a description of the currently available EMIs for depression. As for EMA studies, we will focus both on technological (i.e., devices for intervention delivery, sensors, and biosensors) and technical features (i.e., sampling schemas, duration), as well as on clinical outcomes (i.e., content of the intervention, clinical improvement, patients’ satisfaction/feedbacks, compliance, and dropout rates). This process will help us to identify the key characteristics of a successful intervention, but also highlight possible pitfalls that could be improved.

As systematic review is an iterative process, this result schema could be redefined as the work progresses.

## Discussion

Depression represents an important public health concern, and the development and implementation of new tools is becoming a clinical priority. In that sense, new technologies could lead to the development of innovative assessments with greater ecological validity and higher precision, and to the delivery of more customized interventions to a greater number of people in need. Nevertheless, there still exists a huge gap between the clinical practice and the research field, and the development and use of EMAs and EMIs is mainly confined to laboratory settings.

This systematic review will provide a detailed overview of the state of the art of technology-based EMAs and EMIs for clinically depressed patients. The analysis of both technological, design and clinical aspects will enable us to better identify the advantages and disadvantages of this approach and clarify the research questions opening this review. On the one hand, we will try to identify which clinical fields could actually benefit from the use of technology-based EMAs and EMIs; on the other hand, we will deepen how these tools could be improved. More specifically, we will discuss how the addition of passive data derived from embedded-sensors and wearable biosensors to the traditional self-reports could further foster the potentialities of this approach, and how recent advancements machine learning techniques could fill the current technological gap in EMA and EMI frameworks.

## Additional file

Additional file 1: PRISMA-P checklist. (DOCX 36 kb)

## Abbreviations

BD: Bipolar disorder
BPD: Borderline personality disorder
EMA: Ecological momentary assessment
EMI: Ecological momentary intervention
ESM: Experience sampling method
ICT: Information and Communication Technologies
MDD: Major depressive disorder
